# Gene Selection of Methionine-Dependent Melanoma and Independent Melanoma by Variable Selection Using Tensor Decomposition

**DOI:** 10.3390/genes15121543

**Published:** 2024-11-28

**Authors:** Kenta Kobayashi, Y-h. Taguchi

**Affiliations:** 1 Graduate School of Science and Engineering, Chuo University, Tokyo 112-8551, Japan; 2 Department of Physics, Chuo University, Tokyo 112-8551, Japan; tag@granular.com

**Keywords:** tensor decomposition, methionine, melanoma, A101D, MeWo

## Abstract

Methionine is an essential amino acid. Dietary methionine restriction is associated with decreased tumor growth in preclinical studies and extended lifespans in animal models. The mechanism by which methionine restriction inhibits tumor growth while sparing normal cells is not fully understood. In this study, we applied tensor decomposition-based feature extraction for gene selection from the gene expression profiles of two cell lines of RNA sequencing. We compared two human melanoma cell lines, A101D and MeWo. A101D is a typical cancer cell line that exhibits methionine dependence. MeWo is a methionine-independent cell line. We used the application on R, TDbasedUFE, to perform an enrichment analysis of the selected gene set. Consequently, concordance with existing research on the differences between methionine-dependent melanoma and methionine-independent melanoma was confirmed. Targeting methionine metabolism is considered a promising strategy for treating melanoma and other cancers.

## 1. Introduction

Methionine is an essential amino acid in the human body. Dietary methionine restriction is associated with decreased tumor growth in preclinical studies and extended lifespan in animal models [1]. It must be obtained from dietary sources because the human body cannot produce it internally. The mechanism by which methionine restriction inhibits tumor growth while sparing normal cells is not fully understood, except for the observation that most cancer cells are highly dependent on methionine availability, whereas normal cells can alternate between methionine and homocysteine utilization (methionine independence) [2,3]. In most normal cells, cell proliferation slightly decreases when methionine is limited. However, homocysteine addition to the culture medium restores cell proliferation. Cancer cells typically undergo cell death within 48 to 72 h after the initiation of methionine depletion, with minimal rescue using homocysteine [3]. We conducted a comparison of the methylomes and proteomes of the two human melanoma cell lines. First, A101D represents a typical cancer cell line that exhibits methionine dependence. Secondly, MeWo is a methionine-independent cell line that can be rescued by homocysteine levels [4].

We analyzed gene expression profiles from the high-throughput sequencing dataset GSE225945. Data represent methionine-dependent characteristics of melanoma cells, as measured by RNA-seq. In this analysis, we employ an application called TDbasedUFE [5] to apply variable selection using tensor decomposition. This method has been successfully used for RNA-seq data analysis [6]. Using this method, we selected genes whose expression was independent of replicates and conducted an enrichment analysis to investigate their significant overlap with known gene sets.

## 2. Materials and Methods

### 2.1. Tensor Decomposition

**Figure 1:**
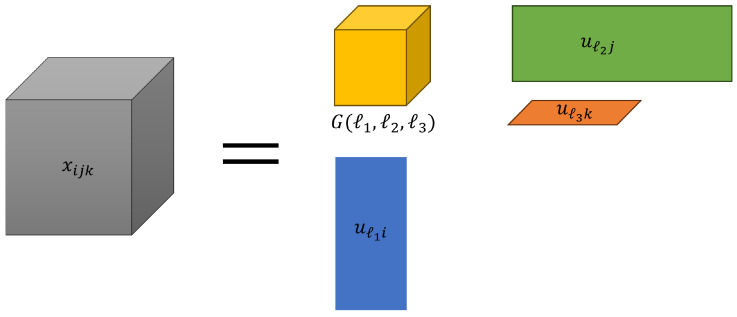
The model of Tucker decomposition.

We employed the Tucker decomposition [7] using the higher-order singular value decomposition (HOSVD) algorithm. Tucker decomposition is a type of tensor decomposition. In the following, we refer to tensor decomposition as TD. An overview of the Tucker decomposition is represented in Figure 1.

This equation expresses Tucker decomposition, which decomposes a tensor into one core tensor and several matrices.

(1) $$\begin{array}{c} x_{ijk}=\sum_{\ell_{1}=1}^{N}\sum_{\ell_{2}=1}^{M}\sum_{\ell_{3}=1}^{K}G\left(\ell_{1},\ell_{2},\ell_{3}\right)u_{\ell_{1}i}u_{\ell_{2}j}u_{\ell_{3}k} \end{array}$$

$x_{ijk}\in R^{N\times M\times K}$ often represents the gene expression of the *i*th gene in the *j*th human subject’s *k*th tissue. In advance, it is necessary to normalize the tensors $x_{ijk}$ such that their mean was centered around zero, and their variance was scaled to one. $G\in R^{N\times M\times K}$ denotes the core tensor, which represents the weight of products $u_{\ell_{1}i}u_{\ell_{2}j}u_{\ell_{3}k}$ to $x_{ijk}$ and $u_{\ell_{1}i}\in R^{N\times N},u_{\ell_{2}j}\in R^{M\times M}$, and $u_{\ell_{3}k}\in R^{K\times K}$ are the singular values and orthogonal matrices, respectively. First, the singular value vector attributed to samples $u_{\ell_{2}j}$ and $u_{\ell_{3}k}$ are analyzed to identify those of interest. Subsequently, the singular value vectors attributed to the genes $u_{\ell_{1}i}$ are selected. Here, $\ell_{1}$ is selected such that the absolute value of *G* is the maximum among identified $\ell_{2}$ and $\ell_{3}$. *p*-values are computed by assuming that $u_{\ell_{1}}$ obeys a Gaussian distribution (null hypothesis) and is adjusted using the Benjamini-Hochberg criterion [7] to consider multiple comparison corrections. As a 2-tailed $\chi^{2}$ test was conducted, the *p*-value is as follows:

(2) $$\begin{array}{c} P_{i}=P_{\chi^{2}}\left[>{\left(\frac{u_{\ell_{1}i}}{\sigma_{\ell_{1}}}\right)}^{2}\right] \end{array}$$

$P_{\chi^{2}}\left[>x\right]$ is the cumulative $\chi^{2}$ distribution, where the argument is larger than *x* and $\sigma_{\ell_{1}}$ is the optimized standard deviation such that $u_{\ell_{1}}$ obeys a Gaussian distribution as much as possible. Finally, *p*-values adjusted to less than the threshold value were selected, and genes were extracted [5,7].

### 2.2. Gene Expression Profile

GSE225945 [8] contains the gene expression profile of methionine dependence on melanoma cells measured by RNA-seq (Supplementary Table S1). The organism for these data was *Homo sapiens*. Data were obtained from the methionine-dependent melanoma cell line A101D and the methionine-independent melanoma cell line, MeWo. Cells were cultured for 24 h in media containing 200 μM methionine or 200 μM homocysteine. These data were available from the Gene Expression Omnibus database under the accession number GSE225945.

### 2.3. TDbasedUFE

In the analysis using TDbasedUFE, the following three components are required: Features (gene names or genomic loci), samples (sample names) and values (gene expression levels or scores). The information obtained from GSE225945 was input into the function ’PrepareSummarizedExperimentTensor’ within the TDbasedUFE package. Subsequently, the resulting tensor was decomposed using the previously described method, HOSVD (Higher-Order Singular Value Decomposition). The function ’SelectSingularValueVectorSmall’ from TDbasedUFE was employed to choose singular value vectors because we need the difference in variable *k* as mentioned in the preceding section. This enables the extraction of genetically characterized features.

### 2.4. Enrichment Analysis

Enrichment analysis was employed to analyze the biological data. We can search for probable associations of genes with specific biological phenomena. Enrichment analysis was performed using the gene set described in the previous section. Enrichr [9,10,11] was used for this purpose. Enrichr is a tool that provides significant insights into genes selected by TDbasedUFE. In this study, gene name conversion was performed by using DAVID [12,13] because the obtained gene set was represented in Ensembl Gene ID (Supplementary Table S2).

## 3. Results

### 3.1. Gene Selection

**Figure 2:**
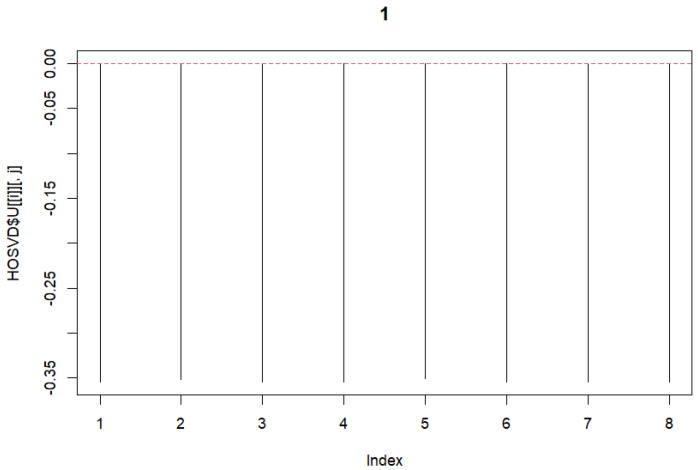
Singular Value Vectors concerning variable *j*.

**Figure 3:**
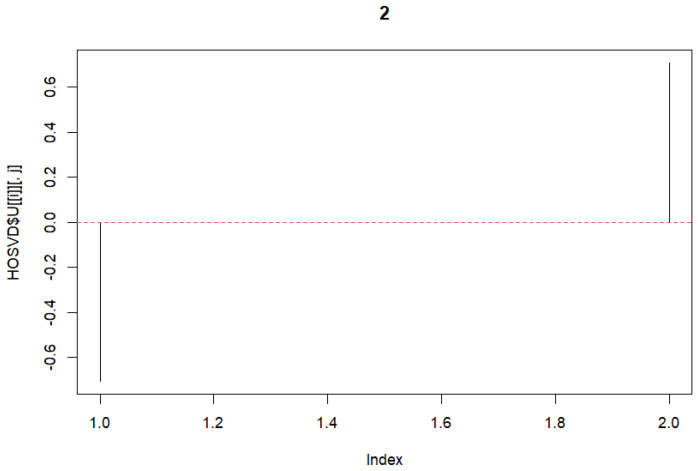
Singular value vectors concerning variable *k*.

HOSVD was performed on the previously mentioned 3rd-order tensor. The tensor with dimensions 66,766×8×2 was formed. Dimension ‘2’ represents A101D and MeWo. Subsequently, singular vectors were selected. First, the vectors for the variable *j* were chosen. As these represent the same, we selected vectors with no differences. Subsequently, vectors related to *k* were chosen. In this case, differences were desired. Therefore, vectors with opposite signs were selected. We selected these and plotted the graphs (Figure 2 and Figure 3).

**Figure 4:**
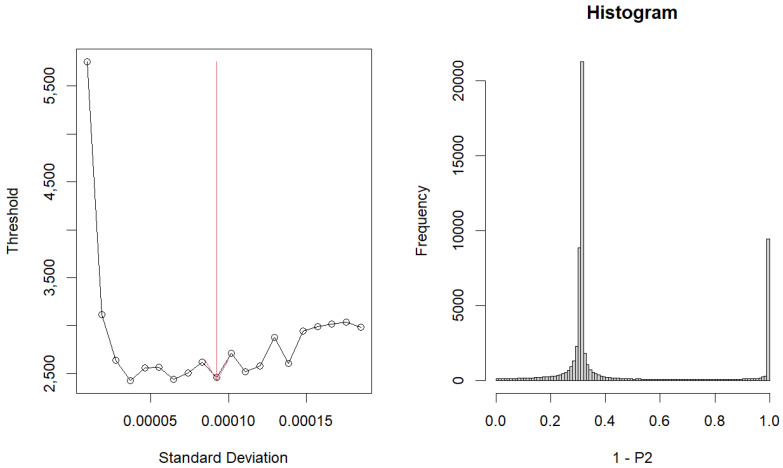
Graph and Histogram about optimized $\sigma_{\ell_{1}}$.

Upon choosing these vectors, the resulting graph was Figure 4. The left graph represents the dependence of the ‘flatness’ of the histogram of adjusted *p*-values. The histogram on the right shows 1−p-values.

As a result, 8656 genes were selected (Supplementary Table S1).

### 3.2. Enrichment Analysis

**Figure 5:**
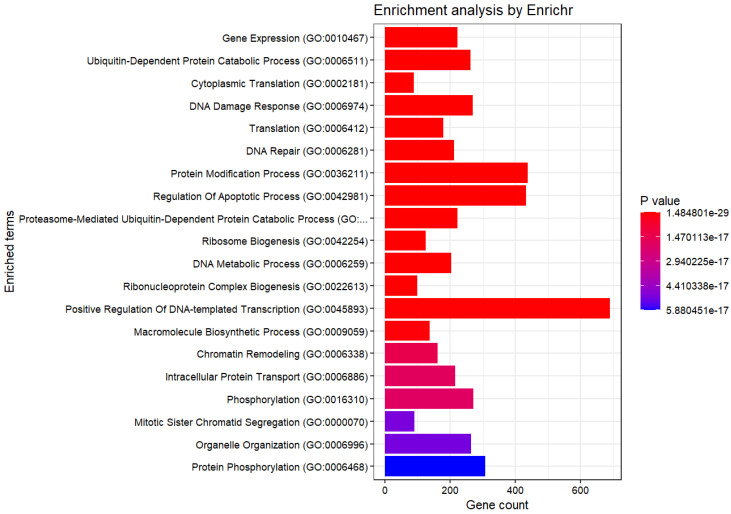
Enrichment Analysis (GO Biological Process 2023).

Subsequently, an enrichment analysis was conducted for the selected genes (Figure 5 and Table 1, Table 2, Table 3, Table 4 and Table 5).

At this level, it can be asserted that all of them have small *p*-values, implying that none of the observations was likely owing to chance. The obtained genes were diverse and numerous, making it difficult to identify the common characteristics.

## 4. Discussion

**Figure 6:**
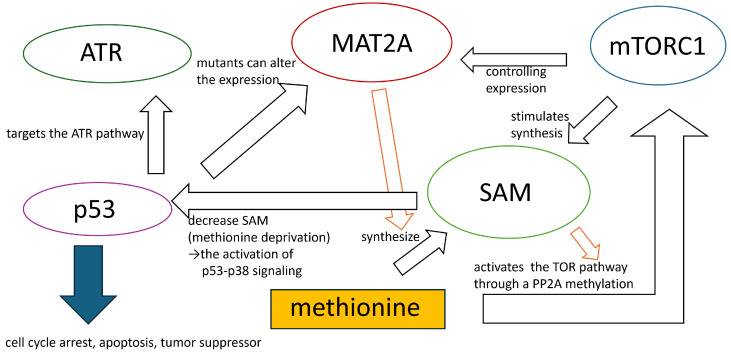
The mechanisms underlying methionine’s influence on tumor cells.

We considered the enrichment analysis results obtained in the Results section. In the current study, methionine-dependent melanoma cells were compared independent melanoma. This allowed us to discuss the differences in methionine content for the two cell lines, GO terms, ‘Gene Expression’, ‘Cytoplasmic Translation’, and ‘Translation’, ‘Protein Modification Process’, from ‘GO Biological Process 2023’ associated with proliferation suggests differences in genes associated with cell proliferation. This was supported by the ‘Cell Cycle Overview’ from the ‘Elsevier Pathway Collection’. Moreover, GO terms such as ‘Ribosome Biogenesis’, ‘Proteasome-Mediated Ubiquitin-Dependent Protein Catabolic Process’, ‘Ribonucleoprotein Complex Biogenesis’, and ‘Phosphorylation’ imply differences in genes involved in protein synthesis and degradation. Additionally, GO terms such as ‘DNA Damage Response’ and ‘DNA Repair’ were suggested differences in genes related to the DNA damage response and repair. The GO term ‘Regulation Of Apoptotic Process’ suggests differences in apoptosis between methionine-dependent and methionine-independent cells. Furthermore, insights from pathways such as ‘Proteins with Altered Expression in Cancer Metabolic Reprogramming’, ‘Metabolic effects of oncogenes and Tumor suppressors in cancer cells’ from ‘Elsevier Pathway Collection’, ‘Control of Gene Expression by Vitamin D Receptor Homo sapiens h vdrPathway’, and ‘Inhibition of Cellular Proliferation by Gleevec Homo sapiens h Gleevec pathway’ in ‘BioCarta 2016’ indicate differences in genes associated with tumor growth inhibition between methionine-dependent and methionine-independent melanoma cells. Moreover, the pathway from the ‘Elsevier Pathway Collection’ and ‘Breast Cancer’ suggests distinctions in genes related to breast cancer. ‘Direct p53 effectors Homo sapiens 67c3b75d-6191-11e5-8ac5-06603eb7f303’ and ‘p53 pathway Homo sapiens a0de862d-6194-11e5-8ac5-06603eb7f303’ from ‘NCI-Nature 2016’ demonstrated differences in the expression of genes associated with p53. The term ‘ATR signaling pathway Homo sapiens 8991cbac-618b-11e5-8ac5-06603eb7f303’ observed in ‘NCI-Nature 2016’ indicates differences in genes associated with ATR. Translation, the cell cycle, DNA damage repair, and apoptosis are important in methionine stress for melanomas [8]. Methionine dependence of melanoma affects cell proliferation by regulating several pathways, including the cell cycle, DNA damage repair, translation, nutrient sensing, oxidative stress, and immune function [8]. Additionally, methionine deprivation lowers glutathione levels and increases oxidative stress, resulting in increased DNA damage and impairment and DNA repair [14]. For example, methionine restriction induces cell cycle arrest, apoptosis, and autophagy in melanoma cells while enhancing the antitumor immune response [8,15]. Methionine stress eliminates the mitotic activity in melanoma cells and induces apoptosis [15]. Loss of mitosis in tumor cells is associated with a marked reduction in cyclin-dependent kinase (*CDK1*) transcription and/or loss of its active form (*CDK1-P-Thr 161*), which coincides with upregulation of *CDKN1A*, *CDKN1B*, and *CDKN1C*. Expression of the proapoptotic *LITAF*, *IFNGR*, *EREG*, *TNFSF/TNFRSF10* and *TNFRSF12*, *FAS*, and *RNASEL* is primarily up-regulated/induced in cells undergoing apoptosis [15]. Although the genes controlling mitotic arrest and/or apoptosis in response to low extracellular methionine levels are unknown, it is likely that such control is exerted via the induction/upregulation of tumor suppressor/growth inhibitor genes such as *TGFB*, *PTEN*, *GAS1*, *EGR3*, *BTG3*, *MDA7*, the proteoglycans (*LUM*, *BGN*, *DCN*), and the downregulation or the loss of function of pro-survival genes, such as *NFkB*, *MYC*, and *ERBB2* [15]. Many genes identified in this study were selected. Methionine depletion in culture media drastically suppressed protein synthesis in immortalized cell lines by impairing recognition of translation start sites [16]. Methionine metabolism affects the synthesis of polyamines, small cationic molecules that modulate the stability and structure of DNA and RNA, thus influencing gene expression and protein synthesis [17,18]. The relationship between the breast cancer and methionine depletion has been shown [19,20,21]. Methionine deprivation suppresses triple-negative breast cancer metastasis in vitro and in vivo [22]. Methionine dependence in melanoma is influenced by the expression and activity of enzymes involved in methionine metabolism, such as methionine adenosyltransferase 2 alpha (MAT2A), which synthesizes s-adenosylmethionine (SAM) from methionine. SAM is a universal methyl donor modulating gene expression, chromatin structure, and signaling pathways [8,16,19,23]. It has been suggested that the mechanistic target of rapamycin complex 1 (mTORC1) is implicated in methionine-dependent processes, as inferred from the term ‘mTORC Signaling’ in the MSigDB Hallmark 2020. mTORC1 stimulates the synthesis of the major methyl donor, SAM, by controlling MAT2A expression [23]. Amino acid restriction can inhibit mTORC1 activity, resulting in autophagy activation [24]. Autophagy is acutely induced in exclusively methionine-limited cells [25,26]. Subsequent studies have shown that methionine activates the TOR pathway through a SAM-mediated PP2A methylation [25,27]. The ATR-initiated signaling pathway halts the cell cycle [28]. p53 is a tumor suppressor protein [28]. Upon activation, p53 targets the ATR pathway to initiate gene transcription involved in cell-cycle arrest and apoptosis. Simultaneously, it regulates the expression of other genes, thereby facilitating cell-cycle control and apoptosis [28]. A decrease in SAM owing to methionine deprivation leads to the activation of p53-p38 signaling [29]. Methionine metabolism affects the stability and function of p53 post-translational modifications such as acetylation and methylation [30]. p53 mutants can alter the expression of methionine metabolism enzymes such as MAT2A [29]. High methionine concentrations suppress the expression of native, but not mutated, p53 [31]. These inhibitory effects are partly owing to the inhibition of cellular growth proliferation, likely via a p53-dependent pathway [31]. Figure 6 illustrates this mechanism. However, the exact mechanism and significance of ATR activation are not fully understood and require further investigation.

**Figure 7:**
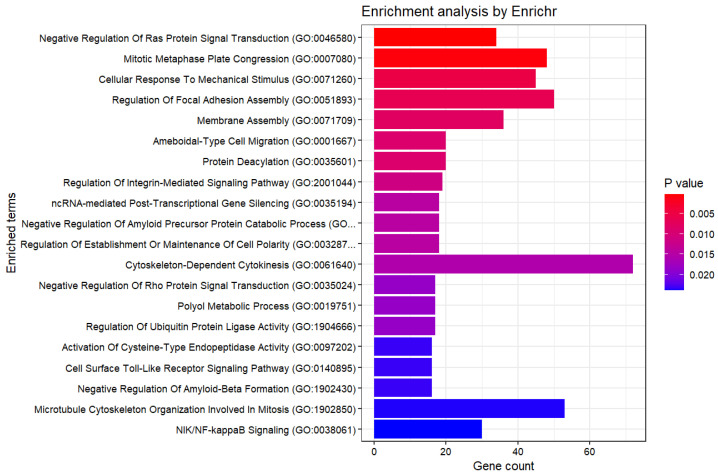
Enrichment Analysis (GO Biological Process 2023, DESeq2).

Using DESeq2 [32] to extract genes with adjusted *p*-values below 0.05 between the two groups, 24,204 genes were selected. Furthermore, enrichment analysis yielded the following results (Figure 7). Compared to our method, a larger number of genes were selected. The *p*-values in the enrichment analysis were also higher than those obtained with our method. Therefore, it can be said that our method is good.

Therefore, the described method for comparing methionine-dependent and methionine-independent melanoma further substantiates these observations. Additionally, various cellular processes are crucial in the cellular response to methionine stress in melanoma. The methionine dependence of melanoma is a complex and dynamic process that affects cell proliferation and survival. Hence, the increased number of selected genes compared to conventional cases may be attributed to a multitude of factors related to differences in the methionine dependence of melanomas.

However, the insights that can be derived from this method are limited. In this study, we drew connections based on previously established knowledge. The abundance of the obtained information makes it challenging to understand what is involved.

## 5. Conclusions

This study is positioned to support the findings obtained from research such as [8] and provide further insights. In summary, we applied HOSVD to the tensor derived from GSE225945 to extract genes. Enrichment analysis of the resultant genes convincingly demonstrated the biological significance level for selected genes. Therefore, the effectiveness of this methodology is evident. However, given the challenges in interpreting the results of this study, exploring the experiments under narrow conditions may provide additional insights. For example, we think it is possible that we applied tensor decomposition-based feature extraction for genes related to antitumor immunity among A101D and MeWo. Therefore, targeting the methionine metabolism is a promising strategy for treating melanoma and other cancers.

## Figures and Tables

**Table 1:** Enrichment Analysis table (GO Biological Process 2023).

Term	Overlap	Adjusted *p*-Value
Gene Expression (GO:0010467)	223/296	$7.91\times 10^{-26}$
Ubiquitin-Dependent Protein Catabolic Process (GO:0006511)	262/367	$1.07\times 10^{-24}$
Cytoplasmic Translation (GO:0002181)	89/93	$1.86\times 10^{-24}$
DNA Damage Response (GO:0006974)	270/384	$4.03\times 10^{-24}$
Translation (GO:0006412)	179/234	$2.67\times 10^{-22}$
DNA Repair (GO:0006281)	212/292	$6.53\times 10^{-22}$
Protein Modification Process (GO:0036211)	437/711	$2.02\times 10^{-20}$
Regulation Of Apoptotic Process (GO:0042981)	432/705	$7.45\times 10^{-20}$
Proteasome-Mediated Ubiquitin-Dependent Protein Catabolic Process (GO:0043161)	222/319	$6.10\times 10^{-19}$
Ribosome Biogenesis (GO:0042254)	125/155	$6.22\times 10^{-19}$
DNA Metabolic Process (GO:0006259)	203/288	$2.53\times 10^{-18}$
Ribonucleoprotein Complex Biogenesis (GO:0022613)	100/118	$4.95\times 10^{-18}$
Positive Regulation Of DNA-templated Transcription (GO:0045893)	690/1243	$1.06\times 10^{-16}$
Macromolecule Biosynthetic Process (GO:0009059)	138/183	$2.75\times 10^{-16}$
Chromatin Remodeling (GO:0006338)	162/228	$4.98\times 10^{-16}$
Intracellular Protein Transport (GO:0006886)	216/325	$6.03\times 10^{-15}$
Phosphorylation (GO:0016310)	272/429	$6.03\times 10^{-15}$
Mitotic Sister Chromatid Segregation (GO:0000070)	91/111	$1.43\times 10^{-14}$
Organelle Organization (GO:0006996)	265/418	$1.43\times 10^{-14}$
Protein Phosphorylation (GO:0006468)	308/500	$1.55\times 10^{-14}$

**Table 2:** Enrichment Analysis table (Elsevier Pathway Collection).

Term	Overlap	Adjusted *p*-Value
Cell Cycle Overview	91/107	$6.35\times 10^{-16}$
Brest Cancer	91/108	$1.16\times 10^{-15}$
Pancreatic Ductal Carcinoma	94/1117	$1.14\times 10^{-15}$
Melanoma	110/145	$5.14\times 10^{-13}$
Proteins with Altered Expression in Cancer Metabolic Reprogramming	72/85	$1.05\times 10^{-12}$
Metabolic Effects of Oncogenes and Tumor Suppressor in Cancer Cells	60/68	$3.41\times 10^{-12}$
Hepatocellular Carcinoma	88/112	$5.41\times 10^{-12}$
Chronic Myeloid Leukemia	61/70	$5.59\times 10^{-12}$
Endometrioid Endometrial Cancer	75/92	$9.71\times 10^{-12}$
Protein Involved in Melanoma	160/238	$1.01\times 10^{-11}$

**Table 3:** Enrichment Analysis table (BioCarta 2016).

Term	Overlap	Adjusted *p*-Value
HIV-1 Nef: negative effector of Fas and TNF *Homo sapiens* h HivnefPathway	43/51	$4.20\times 10^{-6}$
Control of Gene Expression by Vitamin D Receptor *Homo sapiens* h vdrPathway	25/27	$1.12\times 10^{-5}$
Influence of Ras and Rho proteins on G1 to S Transition *Homo sapiens* h RacCycDPathway	25/28	$4.04\times 10^{-5}$
Mechanism of Gene Regulation by Peroxisome Proliferators via PPARa *Homo sapiens* h pparaPathway	40/52	$4.04\times 10^{-5}$
Ceramide Signaling Pathway *Homo sapiens* h ceramide Pathway	28/33	$4.04\times 10^{-5}$
Integrin Signaling Pathway *Homo sapiens* h integrin Pathway	28/33	$4.04\times 10^{-5}$
Cell Cycle: G1/S Check Point *Homo sapiens* h g1 Pathway	23/26	$7.50\times 10^{-5}$
Inhibition of Cellular Proliferation by Gleevec *Homo sapiens* h Gleevec pathway	20/22	$1.20\times 10^{-4}$
Skeletal muscle hypertrophy is regulated via AKT/mTOR pathway *Homo sapiens* h igf1mtor pathway	22/25	$1.20\times 10^{-4}$

**Table 4:** Enrichment Analysis table (MSigDB Hallmark 2020).

Term	Overlap	Adjusted *p*-Value
E2F Targets	180/200	$1.45\times 10^{-42}$
G2-M Checkpoint	176/200	$6.24\times 10^{-39}$
Myc Targets V1	176/200	$6.24\times 10^{-39}$
Mitotic Spindle	172/199	$6.98\times 10^{-36}$
mTORC1 Signaling	166/200	$3.27\times 10^{-30}$
Oxidative Phosphorylation	163/200	$6.32\times 10^{-28}$
Unfolded Protein Response	98/113	$5.25\times 10^{-21}$
UV Response Dn	116/144	$2.50\times 10^{-19}$
Adipogenesis	148/200	$4.54\times 10^{-18}$

**Table 5:** Enrichment Analysis table (NCI-Nature 2016).

Term	Overlap	Adjusted *p*-Value
Direct p53 effectors *Homo sapiens* 67c3b75d-6191-11e5-8ac5-06603eb7f303	103/136	$2.47\times 10^{-12}$
ErbB1 downstream signaling *Homo sapiens* 30d60550-6192-11e5-8ac5-06603eb7f303	83/105	$6.81\times 10^{-12}$
PDGFR-beta signaling pathway *Homo sapiens* c901a3e4-6194-11e5-8ac5-06603eb7f303	95/128	$7.20\times 10^{-11}$
Signaling events mediated by Hepatocyte Growth Factor Receptor (c-Met) *Homo sapiens* ac39d2b9-6195-11e5-8ac5-06603eb7f303	63/77	$2.09\times 10^{-10}$
Validated targets of C-MYC transcriptional activation *Homo sapiens* 61d3b115-6196-11e5-8ac5-06603eb7f303	64/79	$2.66\times 10^{-10}$
ATR signaling pathway *Homo sapiens* 8991cbac-618b-11e5-8ac5-06603eb7f303	37/39	$2.90\times 10^{-10}$
Signaling events mediated by focal adhesion kinase *Homo sapiens* 8fb80085-6195-11e5-8ac5-06603eb7f303	50/58	$4.26\times 10^{-10}$
p53 pathway *Homo sapiens* a0de862d-6194-11e5-8ac5-06603eb7f303	49/57	$7.46\times 10^{-10}$
Aurora A signaling *Homo sapiens* f131cf8e-618b-11e5-8ac5-06603eb7f303	30/31	$4.99\times 10^{-9}$

## Data Availability

The original data presented in the study are openly available in GEO at GSE225945.

## Supplementary Materials

The following supporting information can be downloaded at: https://www.mdpi.com/article/10.3390/genes15121543/s1: Supplementary Tables S1 and S2. Gene Name.
